# Overcoming a travel burden to high-volume centers for treatment of retroperitoneal sarcomas is associated with improved survival

**DOI:** 10.1186/s12957-019-1728-z

**Published:** 2019-11-04

**Authors:** Robin Schmitz, Mohamed A. Adam, Dan G. Blazer

**Affiliations:** Department of Surgery, Duke University Medical Center, Durham, NC USA

**Keywords:** RPS, Travel distance, Volume outcomes, Survival

## Abstract

**Background:**

Guidelines recommend treatment of retroperitoneal sarcomas (RPS) at high-volume centers. However, high-volume centers may not be accessible locally. This national study compared outcomes of RPS resection between local low-volume centers and more distant high-volume centers.

**Methods:**

Patients treated for RPS were identified from the National Cancer Database (1998–2012). Travel distance and annual hospital volume were divided into quartiles. Two groups were identified: (1) short travel to low-volume hospitals (ST/LV), (2) long travel to high-volume hospitals (LT/HV). Outcomes were adjusted for clinical, tumor, and treatment characteristics.

**Results:**

Two thousand five hundred ninety-nine patients met the inclusion criteria. The LT/HV cohort was younger and more often white (*p* < 0.01). The LT/HV group had more comorbidities, higher tumor grade, and more often radical resections and radiotherapy (all *p* < 0.05). The ST/LV group underwent significantly more R2 resections (4.4% vs. 2.6%, *p* = 0.003). Thirty-day mortality was significantly lower in the LT/HV group (1.2% vs. 2.8%, *p* = 0.0026). Five-year survival was better among the LT/HV group (63% vs. 53%, *p* < 0.0001). After adjustment, the LT/HV group had a 27% improvement in overall survival (HR 0.73, *p* = 0.0009).

**Conclusions:**

This national study suggests that traveling to high-volume centers for the treatment of RPS confers a significant short-term and long-term survival advantage, supporting centralized care for RPS.

## Introduction

Soft tissue sarcomas (STS) are a heterogeneous group of tumors that include more than 100 different entities as described in the World Health Organization classification from 2013 [1]. The estimated incidence of STS is approximately 12,750 new cases in the USA in 2019, with an estimated 5270 deaths [2]. Approximately, 15% of all soft tissue sarcomas arise from the retroperitoneum, and this primary site is one of the most difficult areas to treat, given the proximity to major vascular structures and other organs [3, 4].

The primary treatment modality for localized retroperitoneal sarcoma (RPS) is surgical resection. Accomplishing complete resection with grossly negative margins is crucial and associated with improved long-term survival [5, 6]. In fact, postoperative margin status is one of the strongest predictors of outcome in these patients, with median survival differences as large as 103 vs. 18 months based on gross margin status [6]. Despite best efforts, recurrence rates in these patients can be as high as 50–70%. In an effort to decrease recurrence, various strategies have been employed, including extended resection techniques and perioperative radiation therapy, with promising outcomes [7–9].

Given the surgical complexity and the potential role of multimodal treatment strategies to improve outcomes, evaluation of these patients at high-volume centers with multidisciplinary evaluation is recommended [10]. Recent analyses of the NCDB revealed a higher short- and long-term mortality as well as lower R0 resection rate and higher local recurrence rate in patients treated at low-volume centers [11–13]. Population-based studies from Europe similarly revealed higher local recurrence rates in patients not treated at specialized centers [14, 15]. However, not every patient has the ability to pursue care at high-volume, specialized centers, especially when a long travel distance is associated with specialized care.

The relationship between patients’ travel burden to specialized care and their outcomes is not well described in the literature and unanswered for RPS. Some studies have shown that long travel to high-volume medical centers is associated with improved patient outcomes [16, 17]. Yet, some patients prefer to be treated locally. Arguments for treatment at local, low-volume institutions include the reduced travel time for patients and their families, familiarity with the facilities and providers, and the immediate presence of their local support system.

Given the complexity of the treatment of retroperitoneal sarcomas, we hypothesized that traveling long distance for treatment at high-volume institutions would be associated with improved patient outcomes versus short travel distance to a low-volume center. We used the National Cancer Database (NCDB), a robust national database that includes 70% of all cancer cases in this country, to address this question [18].

## Materials and methods

### Data source and study design

This retrospective analysis from the NCDB was approved by the Duke University Institutional Review Board. Patients who underwent resection of a retroperitoneal sarcoma between 1998 and 2012 were identified by the International Classification of Disease for Oncology, 3rd Edition (ICD-O-3) topography codes: 8800, 8801, 8802, 8810, 8830, 8850, 8851, 8852, 8853, 8854, 8858, 8890, 9120, 9540. We excluded patients with missing travel distance or extent of surgery data. Travel distance in the NCDB is calculated as the distance between the center of the patient’s residential zip code and the address of the treating hospital, using Haversine’s formula [19]. Annual hospital volume was calculated for each hospital as the number of RP cases performed at a hospital per year. Patients treated at multiple hospitals were excluded.

All study endpoints were determined prior to data analysis. Preoperative endpoints included neoadjuvant therapy. Perioperative endpoints included surgical margins, hospital length of stay (LOS), 30-day readmission rate, and 30-day mortality. Long-term postoperative endpoints included administration of adjuvant radiotherapy and overall survival calculated as the time from surgery to death or last contact.

### Statistical analysis

Travel distance to treatment centers and annual hospital volume were divided into quartiles. Overlaying the upper and lower quartiles of travel distance with hospital volume, we identify two groups: (1) short patient travel to low-volume hospitals (ST/LV), (2) long patient travel to high-volume hospitals (LT/HV). The above-described outcomes were compared using the Wilcoxon rank-sum test for continuous variables and chi-square or Fisher exact tests for categorical variables. Multivariable logistic regression and Cox proportional hazards models were employed to examine the association between travel distance and the endpoints while adjusting for clinical, tumor, and treatment characteristics, including patient age, gender, race, insurance status, comorbidities, tumor grade, extent of surgery, and radiation therapy.

A two-sided significance level of < 0.05 was used for all statistical tests. All statistical analyses were performed using SAS 9.4 (SAS Institute, Cary, NC).

## Results

**Fig. 1:**
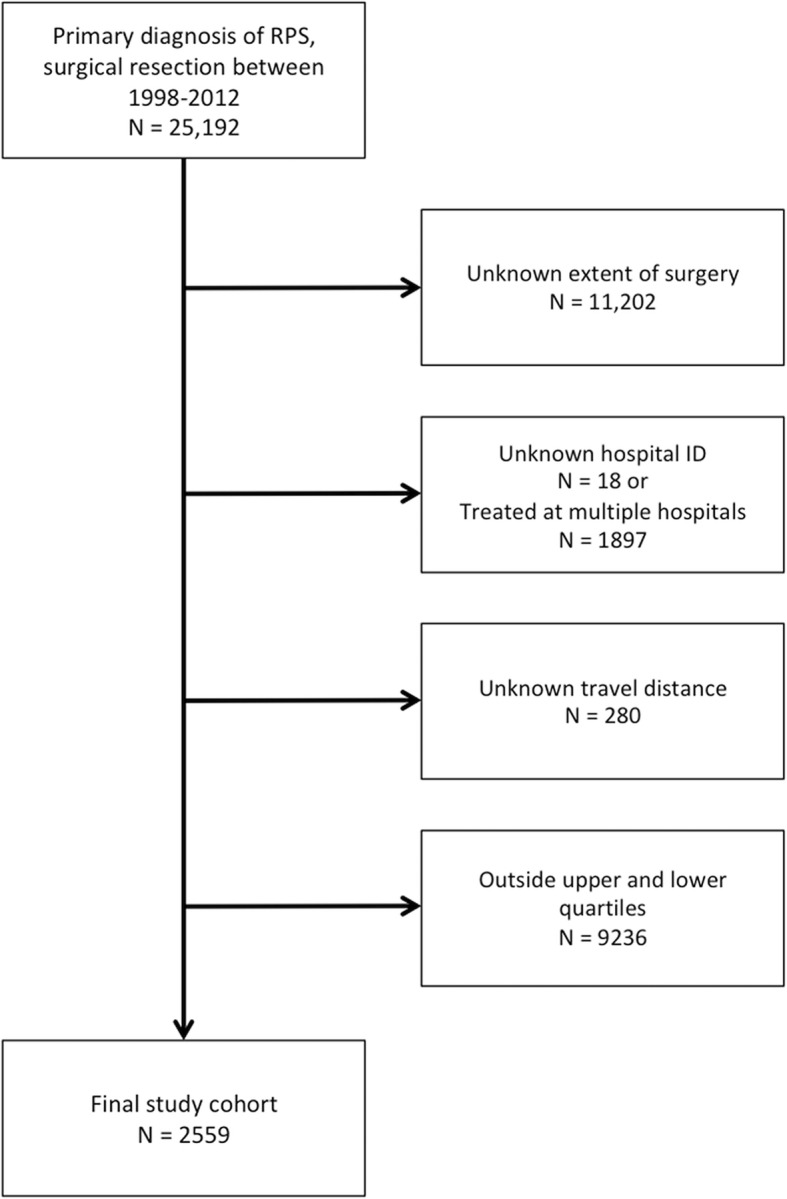
Patient selection and inclusion/exclusion criteria

A total of 2599 patients with retroperitoneal sarcoma were identified in the NCDB between 1998 and 2012 who met inclusion criteria (Fig. 1). Patient characteristics are summarized in Table 1. The median patient age was 61 with a similar gender distribution (51.9% vs. 48.1%). The median travel distance was 14 miles and the median annual hospital RPS case volume was 3 cases/year.

**Table 1:** Characteristics of all included patients from the National Cancer Database who underwent treatment for retroperitoneal sarcomas

	Overall cohort (*N* = 2599)
Age (years, median, IQR)	61 (51–72)
Gender	
Female	1328 (51.9%)
Male	1231 (48.1%)
Race	
Black	233 (9.1%)
Other	122 (4.8%)
White	2204 (86.1%)
Income	
Below median	733 (28.6%)
Above median	1763 (68.9%)
Insurance status	
None	69 (2.7%)
Private/government	2300 (89.9%)
Annual hospital volume (cases, median, IQR)	3 (1–10)
Travel distance (miles, median, IQR)	14 (4–63)
Charlson-Deyo score	
0	1475 (57.6%)
1	319 (12.5%)
≥ 2	77 (3.0%)
Tumor grade	
1	742 (29%)
2	318 (12.4%)
3	1057 (41.3%)
Neoadjuvant therapy	73 (2.9%)
Extent of surgery	
Local	757 (29.6%)
Radical	1011 (39.5%)
Simple	791 (30.9%)
Appropriateness of resection	
R2	89 (3.5%)
R0/1	1722 (67.3%)
Hospital length of stay (days, median, IQR)	6 (3–8)
Radiotherapy	689 (26.9%)
30-day mortality	95 (3.7%)
30-day readmission	95 (3.7%)

One thousand three hundred nine patients were classified as ST/LV group who traveled a median of 4 miles to local medical centers with a median annual case volume of 1 case/year. The second cohort summarized as LT/HV included 1250 patients who traveled a median distance of 56 miles to pursue treatment at high-volume institutions with a median annual case volume of 10 cases/year.

Patients in the LT/HV group were significantly younger and more frequently white (*p* < 0.05). There was no significant difference in the patients’ income and insurance status between the two groups.

In regard to disease-specific characteristics, LT/HV patients had significantly more high-grade sarcomas (*p* = 0.024). Furthermore, patients in the LT/HV cohort underwent more frequent radical resections, defined as resection of other organs in continuity to the primary tumor (51.1% vs. 28.4%), and received more perioperative radiotherapy (all *p* < 0.05). ST/LV patients had significantly more R2 resections (4.4% vs. 2.6%, *p* = 0.003, Table 2).

**Table 2:** Unadjusted characteristics of patients who underwent surgical treatment of retroperitoneal sarcomas at local, low-volume centers (ST/LV) and patients traveling long distance to high-volume centers (LT/HV)

	LT/HV (*N* = 1250)	ST/LV (*N* = 1309)	*p* value
Patient age (years, median, IQR)	59 (50–69)	64 (52–75)	< 0.0001
Gender			0.011
Female	681 (54.5%)	647 (49.4%)	
Male	569 (45.5%)	662 (50.6%)	
Race			0.009
Black	92 (7.4%)	141 (10.8%)	
Other	64 (5.1%)	58 (4.4%)	
White	1094 (87.5%)	1110 (84.8%)	
Income			0.071
Below median	375 (30%)	358 (27.4%)	
Above median	832 (66.6%)	931 (71.1%)	
Insurance status			0.393
None	28 (2.2%)	41 (3.1%)	
Private/government	1057 (84.6%)	1243 (95%)	
Annual hospital volume (cases, median, IQR)	10 (7–14)	1 (1–2)	< 0.0001
Travel distance (miles, median, IQR)	56 (19–132)	4 (2–11)	< 0.0001
Charlson-Deyo score			0.017
0	940 (75.2%)	535 (40.9%)	
1	192 (15.4%)	127 (9.7%)	
≥ 2	39 (3.1%)	38 (2.9%)	
Tumor grade			0.024
1	368 (29.4%)	374 (28.6%)	
2	146 (11.7%)	172 (13.1%)	
3	570 (45.6%)	487 (37.2%)	
Neoadjuvant therapy	61 (4.9%)	12 (0.9%)	< 0.0001
Extent of surgery			< 0.0001
Local	193 (15.4%)	564 (43.1%)	
Radical	639 (51.1%)	372 (28.4%)	
Simple	418 (33.4%)	373 (28.5%)	
Appropriateness of resection			0.003
R2	32 (2.6%)	57 (4.4%)	
R0/1	894 (71.5%)	828 (63.3%)	
Hospital length of stay (days, median, IQR)	6 (4–9)	5 (1–7)	< 0.0001
Radiotherapy	362 (29%)	327 (25)	0.044
30-day mortality	15 (1.2%)	36 (2.8%)	0.0026
30-day readmission	56 (4.5%)	39 (3.0%)	0.38

**Fig. 2:**
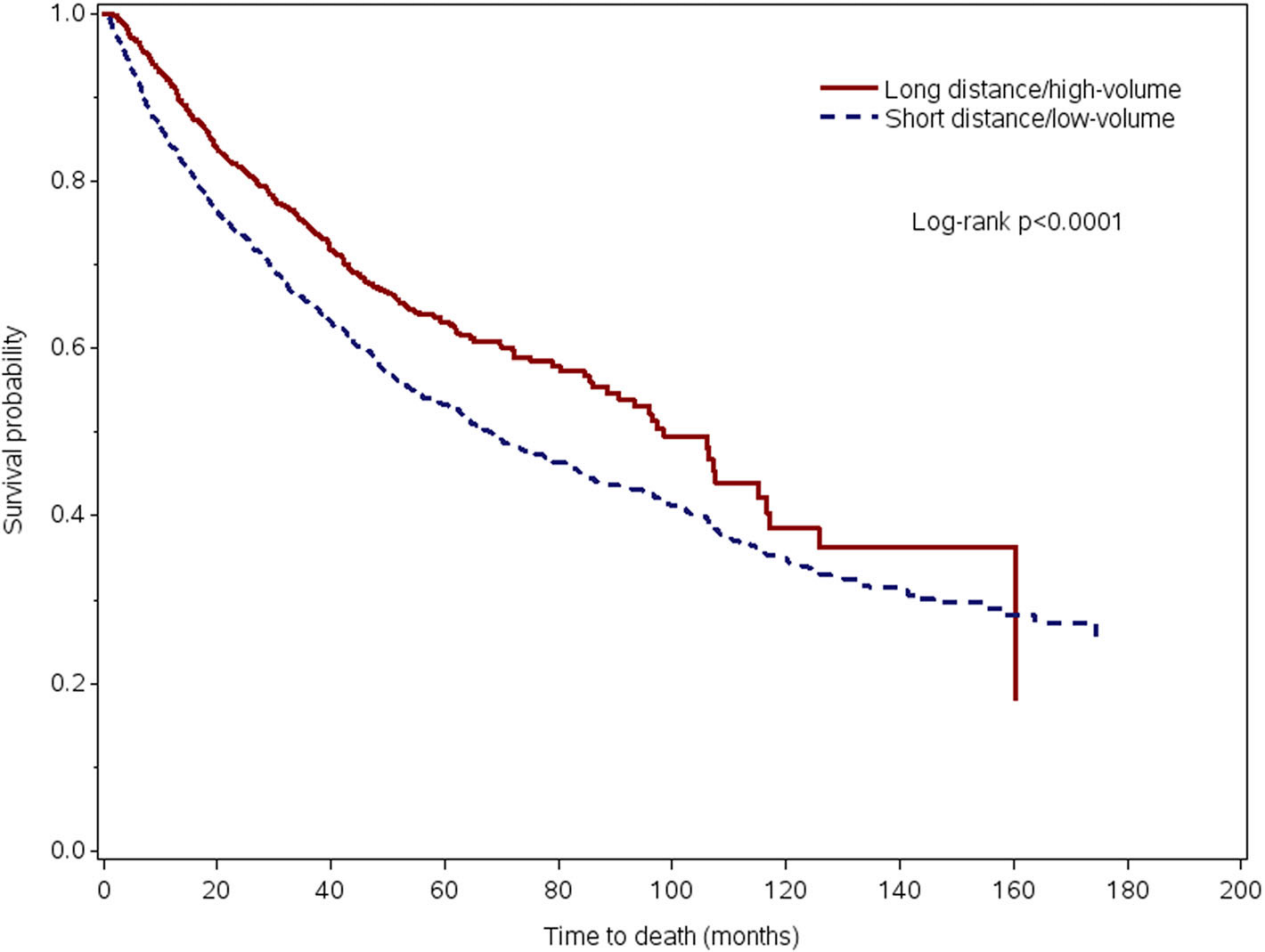
Unadjusted overall survival for patients who traveled to high-volume centers vs. those receiving surgical care at local low-volume centers

No significant difference was identified between groups in the 30-day unplanned readmission rate. Thirty-day mortality was significantly lower in the LT/HV group (1.2% vs. 2.8%, *p* = 0.0026). Five-year survival was better in the LT/HV group (63% vs. 53%, *p* < 0.0001) (Fig. 2), with a median follow-up of 33 months. After adjustment for patient- and disease-specific differences, the LT/HV group had a 27% survival benefit (HR 0.73, *p* = 0.0009) (Table 3).

**Table 3:** Adjusted overall survival for patients who traveled to high-volume centers vs. patients who received low-volume surgical care locally

	Hazard ratio	Lower 95% CI	Upper 95% CI	*p* value
LT/HV^a^ travel	0.726	0.601	0.878	0.0009
Patient age (years, median, IQR)	1.024	1.017	1.031	< 0.0001
Female gender	0.971	0.807	1.168	0.752
Race				
Black	1.030	0.745	1.426	0.857
Other	0.867	0.552	1.362	0.535
Insurance status	0.673	0.356	1.273	0.223
Charlson-Deyo score				
1	1.374	1.100	1.717	0.0052
2	1.328	0.885	1.993	0.171
Tumor grade				
2	1.720	1.202	2.460	0.003
3	3.910	3.049	5.015	< 0.0001
Radiotherapy	0.754	0.615	0.925	0.0068

^a^Long distance travel to high-volume centers

## Discussion

In this nationwide analysis of the NCDB, we investigated the impact of travel distance and hospital case volume on the short- and long-term outcomes of patients with retroperitoneal sarcoma. Given the oftentimes complex and challenging multidisciplinary treatment of RPS, we hypothesized that it remains beneficial for patients to overcome a travel burden to receive care at high-volume centers. This study demonstrates improved short- and long-term outcomes for patients traveling longer distance to high-volume centers. Both 30-day mortality and long-term survival were significantly improved in the group of patients who traveled long distance to high-volume centers versus those patients who stayed closer to home and received care at lower-volume centers. Additionally, patients who traveled short distance to low-volume centers had a significantly higher rate of oncologically inferior R2 resections.

The relationship between hospital volume and improved outcomes is well described in the literature [20–23]. Birkmeyer et al. summarized Medicare data in 2002 that included a total of 2.5 million cases in cardiovascular and cancer surgery. Absolute differences in adjusted mortality rates between low-volume and high-volume centers were as high as 12% for pancreatic resections and as low as 0.2% for carotid endarterectomies [24]. The same group went on to demonstrate improved mortality rates as hospital volumes rose over time [25]. Despite increased awareness and improvements in hospital safety over time, this relationship between higher volume hospitals and improved mortality has remained strong [20–23, 26].

Similar results have also been demonstrated for retroperitoneal sarcomas. In an analysis of the NCDB including more than 5000 patients between 2004 and 2013, patients were divided in 3 groups depending on annual hospital case volume: low volume (< 5 cases/year), medium volume (5–10 cases/year), and high volume (> 10 cases/year). Patients treated at low-volume institutions had a significantly higher risk of 30-day mortality (adjusted OR = 4.66, 95% CI 2.26–9.63) and long-term mortality (adjusted HR = 1.56, 95% CI 1.16–2.11) compared to high-volume institutions [11]. Another study of the NCDB revealed that patients treated at high-volume centers had 2.5-fold higher odds of receiving a R0/R1 resection (*p* = 0.026), and 1.8-fold higher odds of an R0 resection (*P* < 0.001). High volume was defined as the upper 10th percentile in this study [27]. As outlined above, high- and low-volume centers are not clearly defined in the literature, though a few studies have used 10 cases per year as a cut-off [11, 13]. Our group previously failed to define a clear cut off [28]. Thus, in this analysis, we divided the cohort into quartiles to maintain objectivity.

Clearly, the definition of a sarcoma center of excellence goes beyond surgical volume alone. A population-based study from Sweden with 375 patients revealed higher local recurrence rates in patients not treated at a specialized center [14]. A similar study from Great Britain confirmed these results with a significantly higher local recurrence rate at local hospitals (39% vs. 19%) and a small overall survival benefit [15]. A recent analysis of the NCDB showed that resection for RPS performed at academic cancer centers was an independent predictor of margin-negative resection [12]. Finally, in a very compelling analysis from the NETSARC and French Sarcoma Group, investigators demonstrated that patients undergoing surgery in a network of 26 sarcoma reference centers (defined by a multidisciplinary tumor board and expert pathology review) demonstrated improved overall and relapse-free survival rates [29].

Despite compelling data demonstrating improved outcomes at more experienced sarcoma centers, patients still choose to pursue care locally. In a novel analysis by Finlayson et al. evaluating patient preferences surrounding pancreatectomy, 45% of patients were willing to accept double the operative mortality risk in order to stay locally [30]. In addition, numerous hypothetical advantages may accrue to patients who receive treatment locally: reduced travel time for patients and their families, familiarity with the facilities and providers, and the immediate presence of their local support system [30]. These advantages may be particularly important in postoperative care if close follow-up or readmission is needed. In addition, neoadjuvant or adjuvant therapy, as may be recommended for some patients with retroperitoneal sarcoma, may only be feasible for patients locally, due to financial and social support reasons. However, in fact, our analysis demonstrates that patients in the ST/LV group were less likely to receive perioperative radiotherapy. Though it is possible that radiotherapy was given locally and underreported to the NCDB, similar results have been observed for pancreatic cancer and colon cancer, where patients received significantly less frequent adjuvant therapy at low-volume centers [17, 31].

Despite these hypothetical advantages of pursuing treatment closer to home, our data strongly support travel to higher-volume centers for the management of resectable RPS. Short-term operative mortality was improved despite more radical resections. Decreased rates of R2 resections were accomplished in high-volume centers. These outcomes likely reflect greater surgeon experience with these rare, complex malignancies, better perioperative care for these patients, and availability of other subspecialty expertise. Longer-term oncologic outcomes were also improved despite higher-grade tumors in the LT/HV group (HR 0.73, *p* = 0.0009). These findings may be attributed to decreased R2 resection rates but may also reflect better multidisciplinary care, including consideration of perioperative radiation therapy, closer oncologic surveillance, and consideration of novel clinical trials.

From the NCDB, we are unable to reliably explore what level of information is available to patients considering resection for RPS (tumor board discussion, surgical volume, multidisciplinary expertise in soft tissue sarcoma). In the future, these data will likely inform hospital-level accreditation and should inform patient decision-making more strongly.

This analysis has limitations. It is a retrospective analysis of a large dataset. Important data are missing, including locoregional and distant recurrence data. However, the NCDB is a well-validated dataset that captures more than 70% of newly diagnosed cancer cases in the USA.

## Conclusion

This study demonstrates the advantages and importance of patients pursuing care at high-volume institutions, regardless of travel distance involved. We demonstrate both improved short- and long-term outcomes, likely reflecting both improved perioperative morbidity and superior oncologic outcomes. It is crucial that referring providers educate their patients about these data when giving recommendations about where to pursue care.

## Data Availability

The datasets used and/or analyzed during the current study are available from the corresponding author on reasonable request.
